# Polar Value Analysis of Corneal Astigmatism in Intrastromal Corneal Ring Segment Implantation

**DOI:** 10.1155/2016/7127534

**Published:** 2016-10-03

**Authors:** Chang Rae Rho, Min-Ji Kim, Choun-Ki Joo

**Affiliations:** ^1^Department of Ophthalmology and Visual Science, Daejeon St. Mary's Hospital, College of Medicine, The Catholic University of Korea, Seoul, Republic of Korea; ^2^Department of Ophthalmology and Visual Science, College of Medicine, The Catholic University of Korea, Seoul, Republic of Korea; ^3^Department of Ophthalmology and Visual Science, Seoul St. Mary's Hospital, College of Medicine, The Catholic University of Korea, Seoul, Republic of Korea

## Abstract

*Purpose.* To evaluate surgically induced astigmatism (SIA) and the average corneal power change in symmetric intrastromal corneal ring segment (ICRS) implantation.* Methods.* The study included 34 eyes of 34 keratoconus patients who underwent symmetric Intacs SK ICRS implantation. The corneal pocket incision meridian was the preoperative steep meridian. Corneal power data were obtained before and 3 months after Intacs SK ICRS implantation using scanning-slit topography. Polar value analysis was used to evaluate the SIA. Hotelling's trace test was used to compare intraindividual changes.* Results.* Three months postoperatively, the combined mean polar value for SIA changed significantly (Hotelling's *T* ^2^ = 0.375; *P* = 0.006). The SIA was 1.54 D at 99° and the average corneal power decreased significantly by 3.8 D.* Conclusion.* Intacs SK ICRS placement decreased the average corneal power and corneal astigmatism compared to the preoperative corneal power and astigmatism when the corneal pocket incision was made at the preoperative steep meridian.

## 1. Introduction

Intrastromal corneal ring segments (ICRS), which were initially designed to correct mild and moderate myopia [1], are currently used to treat corneal ectatic disorders, including keratoconus [2–4]. ICRS of the same or differing sizes have been used [2, 4]. Studies of ICRS implantation have reported the keratometric, refractive, and visual outcomes [5]. The net result was flattening of the cornea with a decrease in keratometric power. The keratometric changes in ICRS implantation are affected by the incision meridian and the size and diameter of the segments. Previous studies were limited in that the spherical and cylindrical components were treated as independent variables. The analysis of surgically induced astigmatism (SIA) requires conversion of the net astigmatism to orthonormal components in dioptric space [6].

Vector analysis research indicated that the maximum reduction in astigmatism was obtained when the incision and segments were placed along the flat topographic meridian [7, 8]. However, the nomogram for choosing the segment size and incision meridian in these previous studies differed from that recommended by the manufacturer.

The present study was performed to determine the SIA and average corneal power change of Intacs ICRS implantation when the pocket incision meridian and segment size were chosen in accordance with the manufacturer's recommendations. We used the polar value method to analyze the SIA for ICRS implantation [6, 9–11].

## 2. Materials and Methods

This retrospective, nonrandomized study assessed 34 eyes in 34 patients diagnosed with keratoconus who underwent insertion of symmetric Intacs SK ICRS by the same surgeon (Choun-Ki Joo) at our hospital. To prevent bias, only the eye operated on first for each patient was included. This study was conducted in compliance with the regulations of the Institutional Review Board of Seoul St. Mary's Hospital, informed consent regulations, and the Declaration of Helsinki.

### 2.1. Examination Protocol

All patients underwent a comprehensive examination preoperatively, including the uncorrected distance visual acuity (UDVA), corrected distance visual acuity (CDVA), manifest refraction, slit-lamp biomicroscopy, Goldmann tonometry, ultrasonic pachymetry, and simulated keratometry (*K*) using scanning-slit topography (Orbscan II; Bausch & Lomb, Rochester, NY). The main outcome measures were the SIA and change in the average corneal power.

### 2.2. Surgery

All surgeries were performed under topical anesthesia by the same experienced surgeon (Choun-Ki Joo). In all cases, two symmetric Intacs ICRS (Addition Technology, Sunnyvale, CA) with an inner diameter of 6 mm were used. The thickness of the Intacs segments and incision location were selected based on the manufacturer's recommended nomogram. The standard surgical protocol described in previous reports was followed [12, 13]. With the patient in the seated position, the corneal limbus was marked at the 0°, 180°, and 270° axes. Next, with the patient lying on the surgical table, the steep keratometric meridian was identified and marked using a Mendez degree gauge (Katena Products, Denville, NJ) with the aid of preplaced reference points. A femtosecond laser was used for corneal tunneling (IntraLase FS60; Abbott Medical Optics, Santa Ana, CA). The patient interface of the laser was docked in the eye and the incision meridian was adjusted to the previously marked steep meridian. The tunnel depth was set at 80% of the thinnest corneal pachymetry in the midperipheral cornea where it was planned to implant the ICRS. A Sinskey hook and dissector were used to open the incision and tunnel. The Intacs segments were inserted into the tunnel with forceps and spatulas. The incision was closed with a single 10-0 nylon suture. Topical antimicrobial and steroidal agents were prescribed postoperatively for 1 month.

### 2.3. Follow-Up Evaluation

Postoperative follow-up visits were scheduled at 1 week and 1 and 3 months and then at the clinician's discretion. The suture was removed at 1 month. The visual acuity, refraction, and corneal topography were checked 3 months postoperatively. Data collection was limited to 3 months postoperatively to isolate the effects of ICRS implantation from the possibility of keratoconus progression [14, 15]. Patients were excluded from the study if the data at 3 months were incomplete or if they received asymmetric segments.

### 2.4. Data Analysis

Pre- and postoperative corneal power data were compared 3 months postoperatively. Polar value analysis was used to analyze the SIA [9, 11, 16]. All corneal astigmatism data were converted to plus power in net cylinder format. The net astigmatism with the magnitude in diopters and direction in degrees was transformed into an orthonormal polar value expressed in diopters. To calculate the postoperative astigmatic polar values, the steepest preoperative meridian was consistently used as a reference. Differences between pre- and postoperative polar values were calculated and compared.

For example, for the preoperative net cylinder *A* at *a* and the postoperative net cylinder *B* at *b*, the preoperative and postoperative astigmatic polar values were defined as follows [11]:

(1) AKP+0preop=A,AKP+45preop=0,AKP+0postop=B·sin2⁡b+90−a−cos2⁡b+90−a,AKP+45postop=B·sin2⁡b+45−a−cos2⁡b+45−a,ΔAKP+0=AKP+0postop−AKP+0preop,ΔAKP+45=AKP+45postop−AKP+45preop.

It is possible to convert a pair of polar values into a conventional notation.

(2) M=±AKP+02+AKP+452α=arctan⁡M−AKPAKP+45.

For example, from the 27-year-old patient, we obtained the following preoperative topographic readings:

(3) K1=63.5 D  in  the  meridian  101°K2=55.2 D  in  the  meridian  11°A=63.5−55.2=8.3 Da=101°Preoperative  net  astigmatism=8.3 D  at  101°Mean  keratometry=59.4 D.

The postoperative topographic test showed the following results at 3 months:

(4) K1=54.9 D  in  the  meridian  98°K2=50.6 D  in  the  meridian  8°B=54.9−50.6=4.3 Db=98°.

In the same way, we obtained the postoperative net astigmatism (4.3 D at 98°) and mean keratometry (52.8 D).

Conversion to astigmatic polar values is as follows:

(5) AKP+0preop=8.3 DAKP+45preop=0AKP+0postop=B·sin2⁡b+90−a−cos2⁡b+90−a=4.3 DAKP+45postop=B·sin2⁡b+45−a−cos2⁡b+45−a=−0.4 DΔAKP+0=AKP+0postop−AKP+0preop=−4.0 D,ΔAKP+45=AKP+45postop−AKP+45preop=−0.4 D.

This pair of polar values was converted into the following conventional net cylinder notation:

(6) M=4.0 Dα=93°.

The surgically induced polar value in the meridian +0 [ΔAKP(+0)] was the meridional power causing a decrease or increase in power along that plane. The surgically induced polar value in the meridian (+45) [ΔAKP(+45)] was the torsional force twisting the astigmatism in a counterclockwise or clockwise direction.

Statistical analyses were conducted using SPSS (ver. 15.0; SPSS, Chicago, IL). As the refractive components are correlated, multivariate statistical analysis using Hotelling's trace test was used to compare the intraindividual changes [10, 11]. In all analyses, *P* < 0.05 was taken to indicate statistical significance. Univariate analysis was used to assess changes in the respective polar values [ΔAKP(+0) and ΔAKP(+45)]. The results are expressed as mean±standard deviations (SD).

## 3. Results

This study included 34 eyes in 34 patients (22 males, 12 females) with a mean age of 27.0 ± 6.7 (range 19–44) years. The thickness of Intacs segment was selected based on the comprehensive nomogram and presurgical guide recommended by the manufacturer. Intacs implantation significantly decreased the average corneal power by 7.1% less than the preoperative Sim *K* (Table 1).

**Figure 1:**
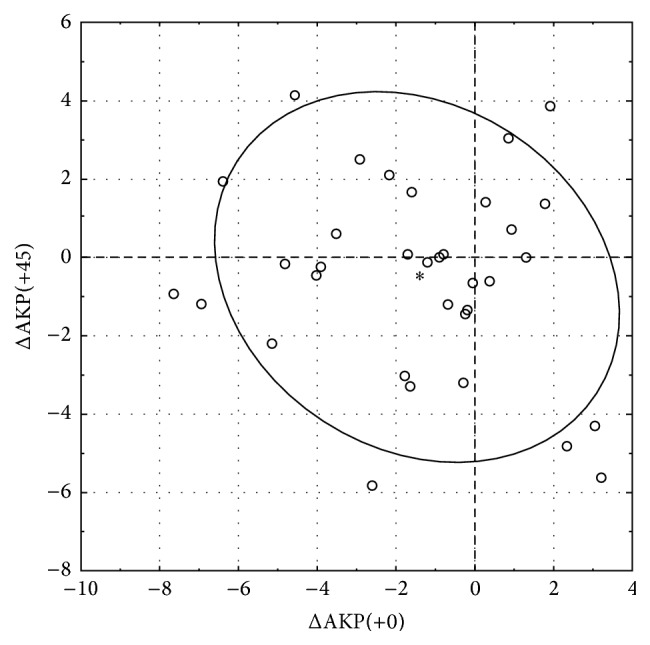
Surgically induced astigmatism is shown as pairs of polar values for the 6 mm Intacs SK implantation. The asterisk denotes the combined mean polar values, and the 95% bivariate confidence ellipse is shown. ΔAKP(+0) = AKP(+0)_postop_ − AKP(+0)_preop_; ΔAKP(+45) = AKP(+45)_postop_ − AKP(+45)_preop_.

Corneal polar value analysis was performed (Table 2). The average corneal power decreased by 1.46 ± 2.80 D and 0.50 ± 2.50 D for ΔAKP(+0) and ΔAKP(+45), respectively. The combined mean polar values for the SIA changed significantly (Hotelling's *T* ^2^ = 0.375; *P* = 0.006). Univariate analysis revealed that AKP(+0) decreased significantly and that the change in AKP(+45), which is the torsional force twisting in the astigmatic direction, was not significant after 3 months. When the results were converted into conventional notation, the SIA was 1.54 D at 99°. The SIA is shown as pairs of polar values in Figure 1. A net decrease in AKP(+0) occurred in 24 eyes (71%).

Subgroup analysis revealed that average corneal power decreased in each thickness subgroup (Table 3). The percentage decreases compared to the preoperative Sim *K* were 4.2, 4.4, and 8.2% in the 350, 400, and 450 *μ*m Intacs subgroups, respectively. The effect was greater in thicker implant groups. Polar value analysis showed that AKP(+0) decreased after 400 and 450 *μ*m Intacs implantation. Statistical analysis was not performed for the subgroups due to the small size of the individual groups.

## 4. Discussion

Intacs are implanted to change the cornea by placing two symmetric or asymmetric segments in the midperiphery of the cornea. In general, symmetric Intacs segments are used for central ectatic cones and asymmetric segments for eccentric ectatic cones [17]. One of the main goals in managing the central cone is to decrease the average corneal power. However, a considerable amount of corneal astigmatism is also found in symmetric cones, and this should be decreased to improve visual quality.

The present study was performed to examine the corneal power and astigmatic effects of 6 mm Intacs SK. In this study, all incisions were located on the preoperative steep meridian and the segment thickness was selected using the nomogram recommended by the manufacturer. The results showed that the average corneal power decreased significantly by 3.8 D after 6 mm Intacs implantation. Corneal astigmatism decreased significantly by polar value analysis.

The keratometric changes observed in this study corresponded with previous reports. In studies of 7 mm Intacs ICRS, the mean change in *K* varied from 1.94 to 4.3 D depending on the author and surgical procedure involved in tunnel creation [12, 14, 18–20]. With regard to the keratometric results for 6 mm Intacs (Intacs SK), Haddad et al. [21] demonstrated that flat and steep keratometry decreased by 1.51 and 2.24 D, respectively. In another study, the mean keratometry (*K*) reading decreased from 52.07 to 46.15 D for *K*1 and from 57.9 to 51.2 D for *K*2 [22]. In a third study of advanced keratoconus, the mean simulated keratometry value decreased from 57.94 to 50.07 D [23]. Intacs SK, which is closer to the visual axis, had a greater flattening effect on the central cornea.

SIA analysis required vectorial analysis that considered the axis of astigmatism. Segments 6 mm in diameter decreased the corneal astigmatism by 1.54 D at 99° from the preoperative steep meridian. Vectorial analysis of ICRS implantation was performed in a few studies [7, 8]. Our results agreed with an earlier study in that the corneal astigmatism decreased after vector analysis. Meanwhile, reports by Tu et al. [7, 8] indicated that the maximum reduction in astigmatism (–2.67 D) occurred when the incision was placed in the flat meridian (perpendicular group) and the least effect (–0.65 D) was when the incision was placed in the steep meridian (meridional group). The differences in study design and analysis make it difficult to directly compare these previous results with those of the present study. First, Tu et al. [7, 8] implanted two symmetric 7 mm Intacs ICRS 0.3 mm thick irrespective of the preoperative keratometric power. In contrast, 6 mm Intacs SK and the manufacturer's nomogram were used in our study. Although decreasing the variables (segment size and diameter) could give a more reliable result, we followed the manufacturer's recommendations for respective patients. Second, the mean keratometric power decreased more in the meridional group (3.93 D) than in the perpendicular group (1.79 D) in the previous study [7]. Symmetric Intacs segments are usually used for keratoconus patients with a relatively central cone. In this situation, decreasing the keratometric power would be the preferred goal of the surgery, rather than decreasing the corneal astigmatism. The meridional group had a better result with regard to decreasing the keratometric power, although the astigmatic effect was less than that in the perpendicular group in Tu et al.'s study. Third, the preoperative keratometric astigmatism itself was greater in the perpendicular group (4.65 D) than in the meridional group (2.40 D), although no statistical preoperative comparison was available.

Our study had a few limitations. First, it was of a retrospective design. Second, the follow-up period was limited to 3 months. Third, we encountered the same problem mentioned in a previous report [7]; that is, the keratometric results were highly variable. The percentage coefficient of variation (SD/mean) was 192% for ΔAKP(+0). This exceeded the value reported by Tu et al., which was 133% for the keratometric surgical effect [14]. We speculate that other variables, including the thickness of the segments, increased the percentage coefficient of variation.

## 5. Conclusion

Our study showed that 6 mm Intacs significantly decreased the corneal power and corneal astigmatism compared to the preoperative values when the segments were implanted according to the recommended nomogram. We demonstrated that locating the incision site on the preoperative steep meridian flattened the steep meridian, expressed as a decrease in AKP(+0). The torsional effect was not significant, as verified by univariate analysis of ΔAKP(+45), which illustrated the accuracy of the incision location. However, the results of our study are readily understood, and the net SIA was clearly shown by the changes in AKP(+0) and AKP(+45). We conclude based on our average corneal power and astigmatic results that the current nomogram recommended by the manufacturer for selection of Intacs thickness and incision location is efficacious.

## Figures and Tables

**Table 1:** Patient demographics.

Parameter	Value
Eyes	34
Male/female	22/12
Age (years)	27.0 ± 6.7
Right/left	20/14
Preoperative keratometry (D)	53.87 ± 5.02
Postoperative keratometry (D)	50.06 ± 4.79
Keratometry change (D)	–3.81 ± 2.09
*P* value	<0.001^*∗*^

^*∗*^Paired-samples *t*-test between preoperative and postoperative keratometry.

**Table 2:** Polar value analysis.

Preoperative polar value (D)		Postoperative polar value (D)		Polar value change (D)		*P* ^*∗*^	*P* ^†^	
AKP(+0)	AKP(+45)	AKP(+0)	AKP(+45)	ΔAKP(+0)	ΔAKP(+45)		ΔAKP(+0)	ΔAKP(+45)
7.29 ± 3.52	0	5.82 ± 4.29	−0.50 ± 2.50	−1.46 ± 2.80	−0.50 ± 2.50	0.006	0.005	0.252

^*∗*^Hotelling's trace between preoperative and postoperative keratometry; ^†^univariate analysis for ΔAKP(+0) and ΔAKP(+45).

**Table 3:** Subgroup analysis.

Thickness (*μ*m)	Number	Preoperative polar value (D)		Postoperative polar value (D)		Polar value change (D)		Preoperative keratometry (D)	Postoperative keratometry (D)	Keratometry change (D)
		AKP(+0)	AKP(+45)	AKP(+0)	AKP(+45)	ΔAKP(+0)	ΔAKP(+45)			
350	3	5.77 ± 2.56	0	6.80 ± 2.42	0.49 ± 1.00	1.03 ± 0.70	0.49 ± 1.00	49.02 ± 0.88	46.93 ± 0.81	−2.08 ± 1.53
400	8	7.33 ± 4.40	0	5.49 ± 4.88	−1.19 ± 1.56	−1.83 ± 1.69	−1.19 ± 1.56	49.93 ± 3.10	47.74 ± 1.86	−2.18 ± 2.00
450	23	7.47 ± 3.40	0	5.81 ± 4.39	−0.39 ± 2.86	−1.66 ± 3.15	−0.39 ± 2.86	55.87 ± 4.65	51.27 ± 5.34	−4.60 ± 1.74
